# Correlation between tomographic scales and vasospasm and delayed cerebral ischemia in aneurysmal subarachnoid hemorrhage

**DOI:** 10.5935/2965-2774.20230119-en

**Published:** 2023

**Authors:** Milagros Gomez Haedo, Pedro Grille, Gastón Burghi, Marcelo Barbato

**Affiliations:** 1 Intensive Care Unit, Hospital Maciel, ASSE - Montevideo, Uruguay

**Keywords:** Vasospasm, intracranial, Subarachnoid hemorrhage, X-ray, computed tomography, Brain ischemia

## Abstract

**Objective:**

To determine the prevalence of sonographic vasospasm and delayed ischemic deficit in patients with aneurysmal subarachnoid hemorrhage, to evaluate the correlation between different tomographic scales and these complications, and to study prognostic factors in this group of patients.

**Methods:**

This was a prospective study of patients admitted to the intensive care unit with a diagnosis of aneurysmal subarachnoid hemorrhage. The prevalence of sonographic vasospasm and radiological delayed cerebral ischemia was analyzed, as was the correlation between different tomographic scales and these complications.

**Results:**

A total of 57 patients were studied. Sixty percent of the patients developed sonographic vasospasm, which was significantly associated with delayed cerebral ischemia and mortality. The Claassen and Hijdra scales were better correlated with the development of cerebral vasospasm (areas under the curve of 0.78 and 0.68) than was Fisher’s scale (0.62). Thirty-two patients (56.1%) developed cerebral infarction on CT; the significantly associated factors were poor clinical grade at admission (p = 0.04), sonographic vasospasm (p = 0.008) and severity of vasospasm (p = 0.015). Only the semiquantitative Hijdra scale was significantly correlated with the development of radiological delayed cerebral ischemia (p = 0.009). The patients who presented cerebral infarction had worse neurological evolution and higher mortality.

**Conclusion:**

This is the first study in our environment on the subject. The Claassen and Hijdra tomographic scales showed better prognostic performance than the Fisher scale for the development of cerebral vasospasm. The finding of sonographic vasospasm could be a noninvasive criterion for the early detection of delayed cerebral ischemia and neurological deterioration in patients with aneurysmal subarachnoid hemorrhage.

## INTRODUCTION

Subarachnoid hemorrhage (SAH) is a devastating neurological disease, accounting for 5% of all strokes.^(1)^ Despite advances in its medical and surgical management, SAH is associated with high mortality and morbidity, with serious sequelae that affect up to half of the patients.^(2-4)^

Delayed cerebral ischemia is one of the main complications of SAH, with an incidence greater than 30%. It is a complex process, the definition of which continues to be debated. Its etiology includes multiple pathophysiological mechanisms, such as early brain injury, vasospasm, inflammation, microthrombosis, alterations in cerebral autorregulation, microcirculation dysfunction, and cortical spreading depression.^(5-10)^

The amount and topography of the blood present in the initial computed tomography (CT) scan constitutes a major risk factor in the development of cerebral vasospasm. Different tomographic scales have been developed that correlate the entity and topography of bleeding with the risk of presenting vasospasm, delayed cerebral ischemia and/or cerebral infarction.^(11-18)^

The objectives of this study were to determine the prevalence of sonographic vasospasm and delayed ischemic deficit in patients with aneurysmal SAH, to evaluate the correlation between the different tomographic scales and these complications, and to study the prognostic factors in this group of patients.

## METHODS

This prospective, single-center study was carried out in an intensive care unit (ICU) of the public health care sector in Uruguay; the center receives approximately 50 patients with SAH annually. The study period was between April 2020 and December 2022. The work was approved by the Ethics Committee of *Hospital Maciel*.

All patients were admitted to the ICU with a diagnosis of SAH of aneurysmal etiology. Patients under 18 years of age, those with an inaccessible or deficient cranial ultrasound window, and those who died within the first 72 hours were excluded. Patients whose initial CT scan was performed after 24 hours from the onset of symptoms were also excluded.

For data collection, the electronic clinical management computer system (Epimed Solutions®) was used, as were audited reviews of the clinical history of each patient, maintaining their confidentiality. The variables recorded were age, sex, clinical grade classification (Hunt and Hess and the World Federation of Neurosurgical Societies - WFNS) at admission, Simplified Acute Physiologic Score 3 (SAPS 3) score on admission, presence of main comorbidities associated with the development of vasospasm, such as high blood pressure, diabetes and smoking, location of the aneurysm, type of treatment (surgical clipping or embolization), length of stay in the ICU, Glasgow Outcome Score (GOS) and mortality at discharge from the ICU and hospital.

All patients were treated in accordance with the institutional protocol for the management of aneurysmal SAH, which included support of vital physiological systems to avoid hypoxemia, hypoand hypercapnia and arterial hypotension, transamine (until the aneurysm was stabilized and for a maximum of 72 hours), enteral nimodipine for 21 days, seizure prophylaxis with phenytoin or valproate for 7 days, and prophylaxis of gastrointestinal bleeding and venous thromboembolism. When vasospasm was associated with ischemic neurological deficit, arterial hypertension was induced with norepinephrine and, if no improvement was observed, endovascular treatment was administered if possible.^(19)^

Tomographic classification was performed using the first CT scan, completed within the first 24 hours of the onset of symptoms. The Fisher, Claassen and Hijdra tomographic scales were used to analyze the CT scan (Table 1).^(11,20,21)^ This evaluation was carried out by 2 researchers (MGH and PG) independently, and in case of disagreement, the opinion of a reference neuroimaging physician was used.^(9,14,15)^

**Table 1:** Tomographic scales for subarachnoid hemorrhage

	Criteria	
Fisher scale^(11)^ (degrees)		
1	No SAH	
2	Diffuse thin layer of SAH < 1mm thickness in vertical cisterns	
3	Clots and/or thick layer of SAH > 1mm in vertical cisterns	
4	Parenchymal or intraventricular clot, with or without diffuse SAH	
Claassen scale^(20)^ (degrees)		
0	No SAH or IVH	
1	Thin layer of SAH, no IVH in both lateral ventricles	
2	Thin layer of SAH, with IVH in both lateral ventricles	
3	Thick layer of SAH^*^, no IVH in both lateral ventricles	
4	Thick layer of SAH^*^, with IVH in both lateral ventricles	
Hijdra Scale^(21)^ (degrees) (0 to 42 points)		
0	No blood	A score of 0 to 3 is awarded for each of:
1	Small amount of blood	- the 10 cisterns and fissures†
2	Moderate amount of blood	- the 4 ventricles‡
3	Full blood filling	

SAH - subarachnoid hemorrhage; IVH - intraventricular hemorrhage.

* Complete filling of one or more cisterns or fissures; † 10 cisternae and fissures: interhemispheric, bilateral sylvian (lateral part), bilateral sylvian (basal part), bilateral suprasellar, bilateral ambiens, quadrigeminal; ‡ 4 ventricles: bilateral frontal, third and fourth.

The velocity of the cerebral blood flow was evaluated by means of blind digital transcranial Doppler (TCD) using a 2 MHz transducer (Digi-Lite TM, Rimed USA, Inc., Long Island City, NY). Both middle cerebral arteries (MCAs) were insonated through the transtemporal window, and both extracranial internal carotid arteries (ICAs) were insonated through the submandibular window. The Lindegaard index was calculated using the mean MCA/ACI velocity ratio.^(10,22-24)^ Sonographic vasospasm was defined as the presence of a mean MCA velocity > 120cm/s and a Lindegaard index > 3. A mean MCA velocity between 120 and 149cm/s was classified as mild, a mean MCA velocity between 150 and 199cm/s was classified as moderate, and a mean MCA velocity ≥ 200cm/s and/or Lindegaard index > 6 was classified as severe.^(25-28)^ All TCD scans were performed under conditions of normocapnia (arterial partial pressure of carbon dioxide - paCO_2_ between 38 and 42mmHg) by the same two experienced operators (MGH and PG). At least two TCD scans were performed for all patients, the first within days 3 to 7 of evolution and the second between days 8 to 12. In the event of vasospasm, scans were repeated on a daily basis, recording higher velocities in the periods mentioned. In the event of clinical neurodegeneration at any time during evolution, ultrasound scans were repeated.

Neurological impairment due to delayed cerebral ischemia was defined as a change in the level of consciousness (decrease in Glasgow Coma Scale - GCS - by 2 or more points) or development of a new focal deficit with a duration of at least 1 hour, from day 3, exhaustively ruling out other causes such as hydrocephalus, rebleeding, metabolic complications, dysnatremia and systemic complications.^(29)^ Cerebral infarction or radiological delayed cerebral ischemia was defined as the presence of cerebral infarction on CT or magnetic resonance imaging (MRI) of the brain within 6 weeks after SAH, not present in the first 48 hours after occlusion of the aneurysm and not attributable to other causes, such as surgical clipping or endovascular treatment. Hypodensities on CT resulting from external ventricular shunt (EVS) placement or the evacuation of parenchymal hematomas were not considered.^(28-30)^

### Statistical analysis

Nominal variables are presented as absolute frequencies or percentages, and continuous variables are presented as medians with interquartile ranges because most data did not present a normal distribution. The comparison of nominal variables was carried out using the chi-square or Fisher test, as appropriate, and continuous variables were compared using the Mann‒Whitney U test. Different tomographic scores were compared based on the development of vasospasm. In this sense, sensitivity, specificity, and positive and negative predictive values were determined, and ROC curves were used to determine the area under the curve. The analysis of factors associated with the GOS was performed by univariate analysis. The significant variables and those clinically relevant were included in a multivariate model after logistic regression. Variables with collinearity were excluded from the model, retaining only one based on clinical relevance. Finally, neurological evolution was evaluated using Kaplan‒Meier survival curves, and the groups were compared using the log rank test. In all cases, p<0.05 was considered significant. SPSS version 21.0 was used for the statistical analyses.

## RESULTS

In the study period, 77 patients were admitted with a diagnosis of aneurysmal SAH, of whom 20 were excluded (12 due to death in the first 72 hours, 7 due to a poor sonographic window and 1 due to loss to follow-up). The demographic and clinical characteristics of the 57 patients studied are shown in table 2. Sixty-five percent of the patients underwent surgical clipping of the aneurysm, and 28% underwent endovascular treatment of the aneurysm.

**Table 2:** Demographic and clinical characteristics of the population

Variables	
Age (years)	52 (45-62)
Female sex	41 (72)
Background information	
High blood pressure	36 (63)
Diabetes	8 (14)
Smoking	31 (54)
SAPS 3	47 (34 - 61.5)
ICU stay (days)	13 (8 - 24)
Mortality in ICU	28 (49)
Mortality in hospital	30 (53)
GOS	4 (3 - 5)
Hunt and Hess classification	
1	5 (8.8)
2	17 (29.8)
3	17 (29.8)
4	6 (10.5)
5	12 (21.1)
WFNS classification	
1	22 (37)
2	9 (16)
3	4 (7)
4	12 (21)
5	10 (17)

ICU - intensive care unit; SAPS 3 - Simplified Acute Physiologic Score 3; GOS - Glasgow Outcome Score; WFNS - World Federation of Neurosurgical Societies. The results are expressed as the median (25th - 75th percentile) or n (%).

Digital arteriography was performed in 22 patients. This procedure was implemented 2.5 days (1 - 6) after admission. Arteriographic vasospasm was defined as a thinning of the contrast medium column in the major cerebral arteries.^(31)^ No significant correlation was found between arteriographic and sonographic vasospasm (Pearson’s correlation: 0.462, p = 0.03) or between arteriographic and radiological cerebral ischemia.

**Figure 1:**
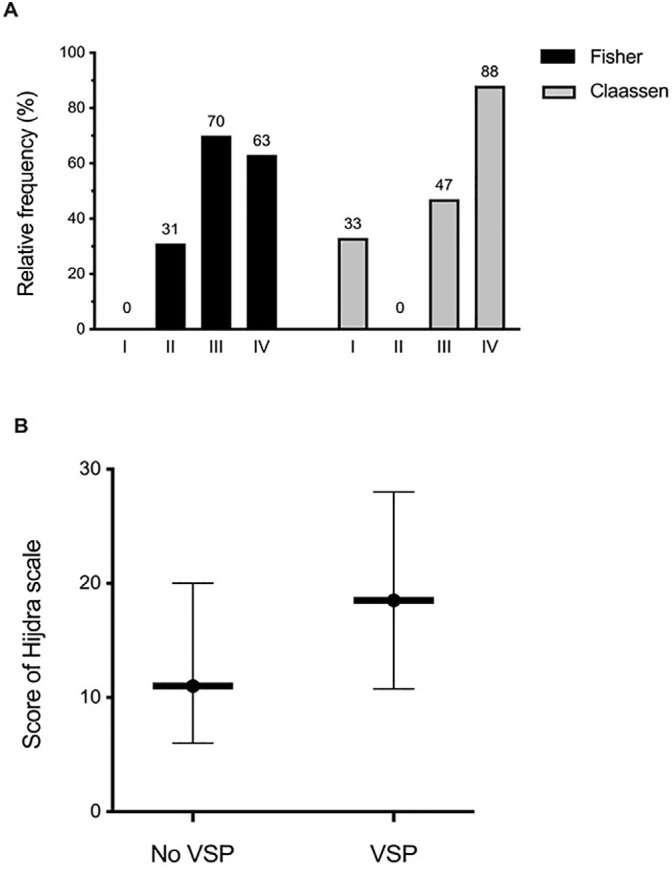
Correlation between tomographic scales and the development of cerebral vasospasm.

**Figure 2:**
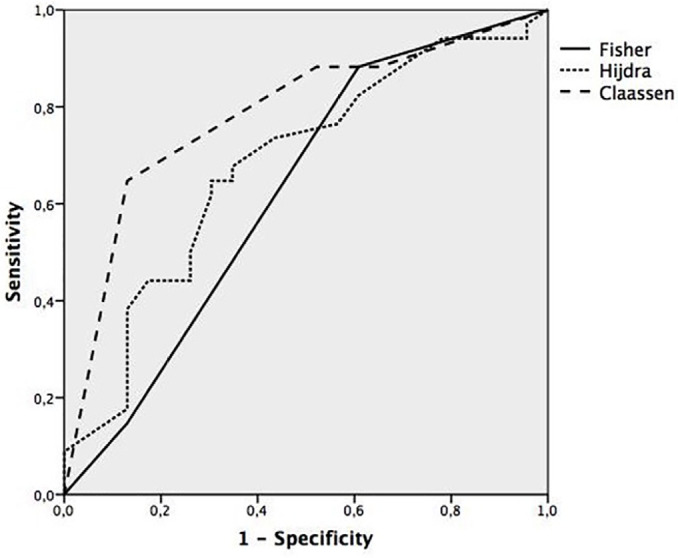
ROC curve showing the correlation between tomographic scales and the development of sonographic vasospasm.

Sixty percent of the patients developed TCD vasospasm. The variables associated with the development of vasospasm are shown in Table 3. Figure 1 shows the correlation between the different tomographic scales and the development of cerebral vasospasm. The Claassen and Hijdra scales were significantly associated with this complication (p = 0.001 and p = 0.022, respectively). Figure 2 shows the ROC curves for the correlations between the tomographic scales and the development of sonographic vasospasm, with the Claassen score showing the greatest area under the curve (0.78), followed by the Hijdra score (0.68) and Fisher’s score (0.62). Category 4 in the Claassen scale showed the highest positive (88%) and negative (63%) predictive value for the development of cerebral vasospasm.

**Table 3:** Factors associated with the development of cerebral vasospasm

	With vasospasm (n = 34)	No vasospasm (n = 23)	p value
Age (years)	50 (44.5 - 62)	56 (49 - 62)	0.51
SAPS 3	47 (34.75 - 58.75)	48 (31 - 63)	0.91
Hunt and Hess classification			0.34
I	2 (40)	3 (60)	
II	10 (59)	7 (41)	
III	8 (47)	9 (53)	
IV	5 (83)	1(17)	
V	9 (75)	3 (25)	
Treatment of the aneurysm			
Clipped	20 (54)	17 (46)	0.24
Endovascular	10 (62)	6 (38)	0.78
Lumbar drainage	19 (73)	7 (27)	0.058
Delayed ischemic deficit	21 (80)	5 (20)	0.004
Cerebral infarction on CT	24 (75)	8 (25)	0.0001
ICU stay (days)	13,5 (9.75 - 18.25)	13 (7 - 28)	0.91
Mortality in ICU	21 (75)	7 (25)	0.02
Hospital mortality	22 (73)	8 (27)	0.02
GOS	4 (3 - 5)	5 (3 - 5)	0.35

SAPS 3 - Simplified Acute Physiologic Score 3; CT - computed tomography; ICU - intensive care unit; GOS - Glasgow Outcome Score. The results are expressed as the median (25th - 75th percentile) or n (%).

A) The relative frequency of cerebral vasospasm is shown for the Fisher and Claassen scales (p = 0.051 and p = 0.001, respectively). B) Hijdra scale scores (expressed as the median and interquartile range) are plotted for patients with and without cerebral vasospasm: 18.5 (10.75-28) versus 11 (6-20), respectively (p = 0.022). VSP - vasospasm.

The areas under the curve were 0.78 (Claassen), 0.68 (Hijdra) and 0.62 (Fisher).

**Figure 3:**
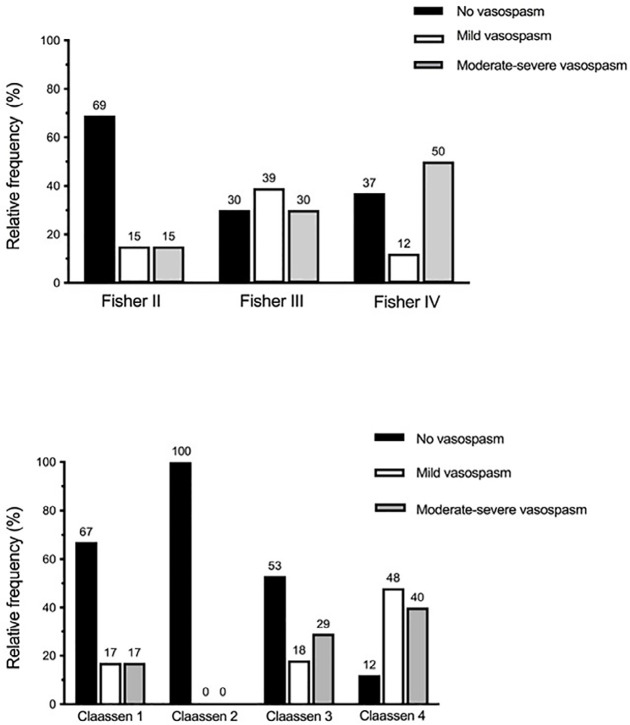
The correlation of the Fisher and Claassen tomographic scales with vasospasm severity.

Regarding the severity of cerebral vasospasm, 5 patients (15%) developed severe vasospasm, 12 (35%) developed moderate vasospasm, and 17 (50%) developed mild vasospasm. Figure 3 shows the correlation of the tomographic scales with vasospasm severity. A score of 4 in the Claassen tomographic classification was significantly associated with the development of moderate or severe vasospasm (p = 0.006).

A score of 4 on the Claassen scale was significantly associated with the development of moderate or severe vasospasm (p = 0.006).

Forty-four patients (81%) were clinically evaluated for neurological deterioration due to delayed ischemia; 25 (57%) were positive for such deterioration. Thirty-two patients (56.1%) presented CT cerebral infarction. The factors that were statistically significantly associated with this presentation were poor clinical grade at admission (p = 0.04), intracranial hypertension (p = 0.013), sonographic vasospasm (p = 0.008), and vasospasm severity (p = 0.015) (Table 4). In 24 (75%) of these patients, sonographic vasospasm was detected, and in those who did not present infarction on CT, sonographic vasospasm occurred in 40%. The maximum mean velocity on TCD for the patients who developed CT infarction was significantly higher than that in those who did not present tomographic infarction: 138 (103 - 158) cm/sec *versus* 84 (66 - 118) cm/sec, respectively; the same occurred with the maximum Lindegaard index value for both groups: 3.6 (2.7 - 4.4) *versus* 2.2 (1.85 - 2.8), respectively. The Fisher and Claassen tomographic scales were not significantly correlated with the development of radiological delayed cerebral ischemia, unlike the Hijdra scale, which was significantly associated with the aforementioned cerebral ischemia (p = 0.009). The patients who presented cerebral infarction had a worse neurological evolution and higher mortality (p < 0.001 and p = 0.001, respectively).

**Table 4:** Factors associated with the development of cerebral infarction or radiological delayed cerebral ischemia

	With cerebral infarction on CT (n= 32)	No cerebral infarction on CT (n= 25)	p value
Age (years)	52.5 (45.25 - 62)	52 (41.5 - 61.5)	0.90
SAPS 3	47.5 (35.75 - 60.2)	44 (31.5 - 63.5)	0.67
Hunt and Hess classification: 3 to 5	13 (40.6)	5 (20)	0.09
WFNS rating: 3 to 5	16 (50)	6 (24)	0.04
Fisher score: 3 and 4	27 (84.3)	17 (68)	0.14
Claassen score: 3 and 4	26 (81.2)	16 (64)	0.14
Hijdra score	18 (11 - 28)	7.5 (5 - 17.25)	0.009
Cerebral vasospasm	24 (75)	10 (40)	0.008
Moderate - severe vasospasm	14 (43.7)	3 (12)	0.015
Lumbar drainage	18 (56)	8 (32)	0.06
Intracranial hypertension^*^	16/26 (61.5)	6/11 (54.5)	0.013
ICU stay (days)	13 (9.25 - 26.5)	14 (7.5 - 26.5)	0.74
Hospital mortality (days)	22 (68.7)	8 (32)	0.006
Hospital mortality	22 (69)	8 (27)	0.02
GOS (1 - 3)	29 (91)	11 (44)	0.001

CT - computed tomography; SAPS 3 - Simplified Acute Physiologic Score 3; WFNS - World Federation of Neurosurgical Societies; ICU - intensive care unit; GOS - Glasgow Outcome Score.

* Intracranial pressure was monitored in only 37 patients. The results are expressed as the median (25th - 75th percentile) or n (%).

**Figure 4:**
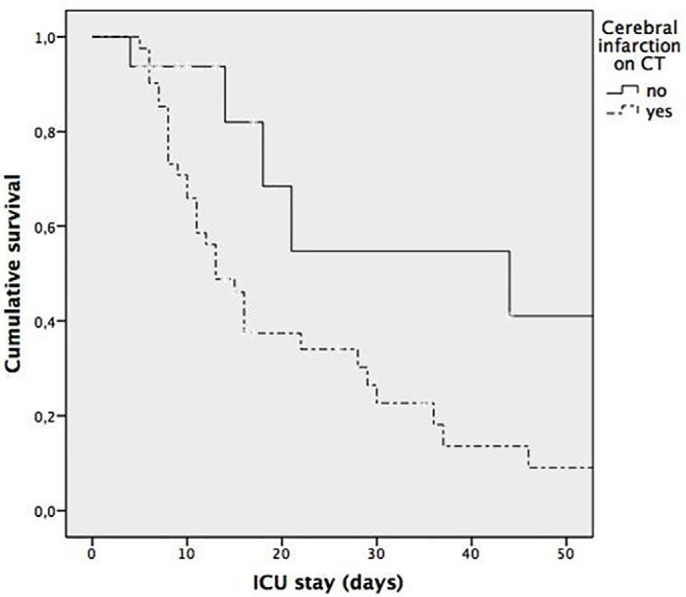
Kaplan-Meier survival curves for patients with and without cerebral infarction on computed tomography (log rank p = 0.012).

Forty patients (70%) had a poor neurological outcome, defined by a GOS of 1 to 3. The causes of death were 77% neurological and 23% nonneurological. Factors associated with poor outcomes were greater clinical severity upon admission (defined by a higher SAPS 3 score and Hunt and Hess and WFNS classifications), intracranial hypertension, a higher score on the 3 tomographic scales and CT cerebral infarction. The multivariate analysis of the factors that did not present collinearity showed that CT cerebral infarction was the only factor that was independently associated with poor neurological evolution (odds ratio - OR 8.2; 95% confidence interval - 95%CI 1.043-64.83) (Table 5). Figure 4 shows the survival curves for patients with and without CT cerebral infarction (log rank p = 0.012).

**Table 5:** Factors associated with poor neurological evolution (Glasgow Outcome Score 1 to 3). Multivariate analysis

	OR	95%CI	p value
High blood pressure	3.5	0.68 - 18.82	0.13
SAPS 3 (for each point)	1.03	0.97 - 1.09	0.29
Cerebral vasospasm	1.14	0.17 - 7.57	0.88
Cerebral infarction on CT	8.2	1.043 - 64.83	0.045
Claassen Score III and IV	1.2	0.198 - 7.66	0.84

OR - odds ratio; 95%CI - 95% confidence interval; SAPS 3 - *Simplified Acute Physiologic Score 3*; CT - computed tomography.

CT - computed tomography; ICU - intensive care unit.

## DISCUSSION

The association between the amount and topography of the blood in SAH with the development of cerebral vasospasm and delayed ischemia has been described in multiple clinical studies.^(15,18,32)^ In this sense, over the years and from the scale originally described by Fisher et al., several tomographic scores have been designed to predict the development of these complications.^(11,20,21,32)^ However, there are discrepancies between the different variables used to quantify these complications as well as the diagnostic accuracy of these scales to predict the complications.^(9,12,33-36)^

This is the first study on the subject in our environment. TCD is routinely used in neurocritical patients as a diagnostic screening tool to identify vasospasm of larger cerebral arteries, with good accuracy compared to digital arteriography, which is the gold standard.^(37-41)^ The incidence of sonographic vasospasm in our population was 60%, a finding that is consistent with the results reported for different series.^(9^.^42-44)^ Delayed cerebral ischemia and evolution in the ICU were the only variables that were significantly associated with vasospasm, reaffirming the concept indicated by other authors that sonographic detection could be implemented as a tool for the clinical detection of neurodeterioration.^(28,45)^

In our study population, the Claassen and Hijdra tomographic scales showed the best correlation with the development of sonographic vasospasm. Consistent with the results reported by Frontera et al., the original Fisher scale, which has been widely used as a prognostic tool, presents weaknesses mainly in cases with a thick layer of subarachnoid blood associated with parenchymal or intraventricular hemorrhage. This has generated confusion in the tomographic staging of patients with SAH, also showing little statistical correlation with the development of cerebral vasospasm in relation to the other scales analyzed.^(13)^

Fifty-six percent of the patients in our series developed radiological delayed cerebral ischemia, a percentage that is somewhat higher than that reported in the literature.^(15)^ Although neurological deterioration due to delayed ischemia is a variable associated with poor neurological evolution in SAH, it is difficult to define objectively. For this reason, in our study, radiological delayed ischemia or cerebral infarction on CT was considered, which represents a part of all these patients but surely includes a subgroup with greater severity.^(28,30)^ Consistent with the results reported for other series, delayed cerebral ischemia was statistically significantly associated with clinical severity upon admission, cerebral vasospasm and vasospasm severity. In 25% of the patients who developed tomographic cerebral infarction, sonographic vasospasm was not detected, a finding that could be due to the existence of other pathogenic factors associated with the development of delayed cerebral ischemia, as has been shown in several clinical studies, such as cortical spreading depression, microthrombosis, neuroinflammation and hypoperfusion due to increased intracranial pressure (ICP).^(15,46)^ Regarding this last factor, although ICP was only monitored in 64.9% of the patients in our series, the presence of intracranial hypertension was significantly associated with the development of cerebral infarction, indicating the possible contribution of cerebral hypoperfusion due to elevated ICP to cerebral ischemia in our patients. Another factor that could also explain this difference is the fact that TCD has very good specificity but moderate sensitivity; therefore, there could be cases of undetected cerebral vasospasm that could affect the development of delayed cerebral ischemia in our population.

In our study, the Fisher and Claassen scales were not significantly correlated with the development of cerebral infarction, unlike the Hijdra tomographic scale. One factor that could explain this is that the Fisher and Claassen scales are qualitative, which is why they have been criticized by different authors due to their lack of reliability linked to sometimes confusing classification criteria.^(47)^ The semiquantitative Hijdra scale allows a more objective assessment of blood volume, with a better prognostic accuracy, which could also explain why in our population the Hijdra scale score, not qualitative scale scores, was significantly associated with radiological cerebral ischemia. Importantly, the measurement of the amount of blood using these 3 scales continues to depend on the observer; therefore, the quantification of the real blood volume on CT using computer programs is the ideal reference method.^(47,48)^

The neurological outcome of our patients reflects the severity of this disease, with 70% of the patients who were discharged from the ICU having a GOS of 1 to 3. Cerebral infarction on CT was the only independent factor associated with severity, with a relative risk of more than 8 times of presenting poor evolution.

This study has several limitations. First, this was a single-center study with a relatively small number of patients, although it was relevant to our environment. Second, the diagnosis of vasospasm was made by TCD because systematic digital arteriography, which is the reference method is not performed in our unit. Third, because the initial CT scan was analyzed to determine scale scores, blood clearance was not taken into account in the evolution, which has been shown to be a positive prognostic factor.^(49)^ Fourth, TCD was followed up until day 12 of evolution, which includes the period of time with the highest incidence of vasospasm, with reports of development until day 21, thus potentially resulting in underdiagnosis.^(28,50)^ Fifth, given that in our institution we do not have CT with perfusion, the incidence of delayed cerebral ischemia may be underestimated.^(51)^ Sixth, 19% of the patients could not be clinically evaluated for neurological deterioration due to their initial severity and/or need for sedation. Finally, the evolution of the patients was followed until discharge from the hospital and not at 6 months, as is recommended for such patients.

## CONCLUSION

This is the first study on this subject in our environment. Our findings indicate that the Claassen and Hijdra tomographic scales show better performance and could be useful prognostic tools for cerebral vasospasm development. Likewise, the finding of sonographic vasospasm can serve as a noninvasive criterion for the early detection of delayed cerebral ischemia and neurological deterioration in patients with subarachnoid hemorrhage. In our population, only the semiquantitative Hijdra scale was correlated with cerebral infarction on computed tomography. Studies with a larger number of patients are needed to confirm these results.
